# Factors Associated with Longitudinal Changes in Mammographic Density in a Multiethnic Breast Screening Cohort of Postmenopausal Women

**DOI:** 10.1155/2023/2794603

**Published:** 2023-10-17

**Authors:** Hannah Lui Park, Argyrios Ziogas, Stephen A. Feig, Roza Lorin Kirmizi, Christie Jiwon Lee, Andrea Alvarez, Rachel McFarland Lucia, Deborah Goodman, Kathryn M. Larsen, Richard Kelly, Hoda Anton-Culver

**Affiliations:** ^1^Department of Pathology and Laboratory Medicine, University of California, Irvine, CA, USA; ^2^Department of Epidemiology and Biostatistics, University of California, Irvine, CA, USA; ^3^Department of Medicine, University of California, Irvine, CA, USA; ^4^Department of Radiological Sciences, University of California, Irvine, CA, USA; ^5^Department of Biological Sciences, University of California, Irvine, CA, USA; ^6^Department of Pharmaceutical Sciences, University of California, Irvine, CA, USA; ^7^Department of Family Medicine, University of California, Irvine, CA, USA; ^8^Department of Clinical Informatics, University of California, Irvine, CA, USA

## Abstract

**Background:**

Breast density is an important risk factor for breast cancer and is known to be associated with characteristics such as age, race, and hormone levels; however, it is unclear what factors contribute to changes in breast density in postmenopausal women over time. Understanding factors associated with density changes may enable a better understanding of breast cancer risk and facilitate potential strategies for prevention.

**Methods:**

This study investigated potential associations between personal factors and changes in mammographic density in a cohort of 3,392 postmenopausal women with no personal history of breast cancer between 2011 and 2017. Self-reported information on demographics, breast and reproductive history, and lifestyle factors, including body mass index (BMI), alcohol intake, smoking, and physical activity, was collected by an electronic intake form, and breast imaging reporting and database system (BI-RADS) mammographic density scores were obtained from electronic medical records. Factors associated with a longitudinal increase or decrease in mammographic density were identified using Fisher's exact test and multivariate conditional logistic regression.

**Results:**

7.9% of women exhibited a longitudinal decrease in mammographic density, 6.7% exhibited an increase, and 85.4% exhibited no change. Longitudinal changes in mammographic density were correlated with age, race/ethnicity, and age at menopause in the univariate analysis. In the multivariate analysis, Asian women were more likely to exhibit a longitudinal increase in mammographic density and less likely to exhibit a decrease compared to White women. On the other hand, obese women were less likely to exhibit an increase and more likely to exhibit a decrease compared to normal weight women. Women who underwent menopause at age 55 years or older were less likely to exhibit a decrease in mammographic density compared to women who underwent menopause at a younger age. Besides obesity, lifestyle factors (alcohol intake, smoking, and physical activity) were not associated with longitudinal changes in mammographic density.

**Conclusions:**

The associations we observed between Asian race/obesity and longitudinal changes in BI-RADS density in postmenopausal women are paradoxical in that breast cancer risk is lower in Asian women and higher in obese women. However, the association between later age at menopause and a decreased likelihood of decreasing in BI-RADS density over time is consistent with later age at menopause being a risk factor for breast cancer and suggests a potential relationship between greater cumulative lifetime estrogen exposure and relative stability in breast density after menopause. Our findings support the complexity of the relationships between breast density, BMI, hormone exposure, and breast cancer risk.

## 1. Introduction

Mammographic breast density is a known risk factor for breast cancer, even after adjustment for other established breast cancer risk factors [1–3]. The association between mammographic breast density and breast cancer has been shown in both premenopausal and postmenopausal women and when breast density is measured quantitatively or categorized qualitatively [1–8]. While quantitative assessments are often limited in large cohort studies because they require a professional observer or specialized software for computer-assisted quantitation and digitized film or processed images, such as Cumulus or Volpara [9–11], qualitative density classifications assigned by radiologists after visual inspection of clinical mammograms according to the American College of Radiology's Breast Imaging Reporting and Data System (BI-RADS) [12] is readily available in large cohorts of patients within clinical settings. The four BI-RADS density categories are (A) almost entirely fat, (B) scattered fibroglandular density, (C) heterogeneously dense, and (D) extremely dense. Studies have shown that an increased BI-RADS density category is associated with breast cancer risk. Women with extremely dense breasts have a greater than three-fold increased breast cancer risk compared to women with breasts that are almost entirely fat [1, 5, 6, 13].

Longitudinal changes in breast density are also associated with breast cancer risk: an increase in mammographic density, even a one-category increase, is associated with increased risk for breast cancer, while a decrease in density is associated with decreased risk compared to women who did not experience any change in breast density [14–17]. For example, in a study of 301,955 women, Kerlikowske et al. showed that women who exhibited an increase in BI-RADS density from category 1 to 2 or 1 to 3 had a risk ratio of 1.9 (95% CI = 1.4–2.6) and 3.4 (95% CI = 2.0–5.7), respectively, compared to women whose BI-RADS density remained in category 1 [15]. Associations between longitudinal changes in breast density and breast cancer risk persisted regardless of age, menopausal status, and family history [16], although they were shown to be more prominent for women aged 50 years and older than for women younger than 50 years of age [15]. In a pooled meta-analysis of five cohort studies, increased breast density over time was associated with higher breast cancer risk (HR: 1.61; 95% CI: 1.33–1.96), whereas decreased breast density over time was associated with lower breast cancer risk (HR: 0.78; 95% CI: 0.71–0.87) [18]. A recent nested case-control cohort study of 10,481 women which used a median of four digital mammograms per participant during a follow-up period of 10 years found that while breast density decreased over time in both cases and controls, women with a slower decrease in breast density had a higher risk of developing breast cancer [19].

Given the connection between longitudinal changes in breast density and breast cancer risk, the identification of factors that are associated with changes in breast density may inform our understanding of breast cancer development and suggest strategies for breast cancer prevention. Cross-sectional studies have shown that breast density is largely genetic [20, 21] but also associated with age and some lifestyle factors. For example, breast density is inversely associated with age, number of children, breast feeding [16, 17], and body mass index [5, 22] and positively associated with alcohol consumption [23, 24] and hormone use [22]. However, there have been only limited studies on factors associated with longitudinal changes in breast density.

Several longitudinal studies have shown that breast density usually declines with age, especially after menopause [15, 19, 25]. High premenopausal density has also been associated with a higher likelihood of reducing breast density across the menopause transition [26], while women with a body mass index over 25 kg/m^2^ experienced slower declines in densities over time, as did the women with Japanese ancestry compared to Caucasian women and women who were taking hormone replacement therapy [25, 26]. To our knowledge, these are the only studies which have examined factors potentially associated with changes in breast density. Identification of additional factors could improve our understanding of breast cancer risk and potentially enable opportunities for enhanced risk assessment and reduction. Given the strong link between longitudinal breast density changes and breast cancer risk, and since the majority of breast cancers are diagnosed in postmenopausal women, the goal of this study was to identify factors associated with longitudinal changes in breast density in a multiethnic cohort of postmenopausal women, using data from a large screening mammography cohort with longitudinal mammographic density measures.

## 2. Methods

### 2.1. Study Cohort

**Figure 1:**
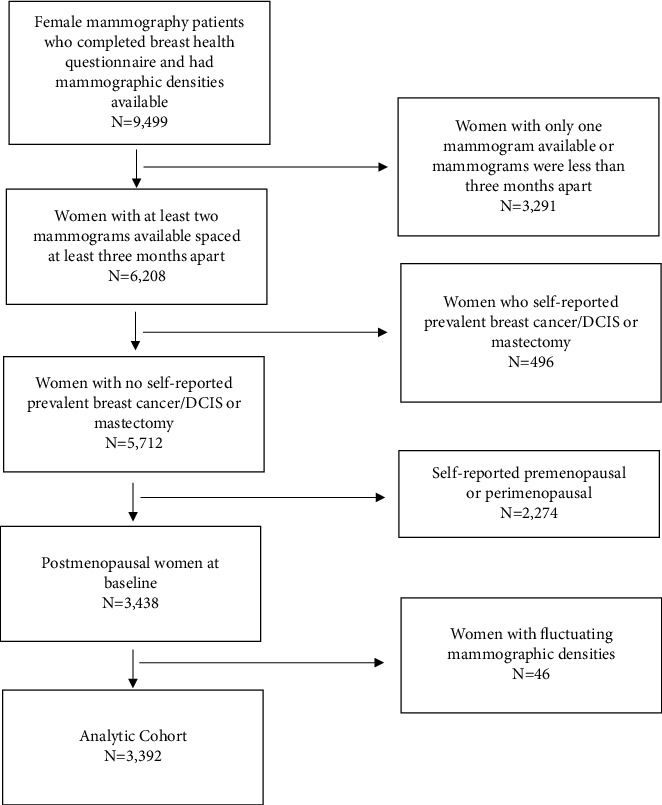
Flowchart with inclusion/exclusion criteria for patients in the analytic cohort.

This study protocol (HS#2010-7489) was approved by the University of California Irvine (UCI) Institutional Review Board (IRB). The study cohort was comprised of female UCI health mammography patients aged 33–92 who completed an electronic clinical intake form prior to their screening mammogram between 2011 and 2017, had at least two mammograms performed at a UCI Health facility spaced at least three months apart, had no history of breast cancer/DCIS or mastectomy, and were postmenopausal at baseline (Figure 1). Women were considered postmenopausal if they reported that their period had naturally stopped or if they had previous removal of both of their ovaries. Since the study outcomes were either increased or decreased longitudinal mammographic density, patients were excluded if they had mammographic densities that both increased and decreased across longitudinal exams (*n* = 46). 3,392 women met these criteria (Figure 1).

### 2.2. Assessment of Mammographic Density Measures

BI-RADS density categories were assigned by radiologists as part of patients' routine clinical mammography screening and retrieved from their UCI electronic health records (EHR). BI-RADS density categories were as follows: almost entirely fat (A), scattered fibroglandular density (B), heterogeneously dense (C), or extremely dense (D). Women were categorized based on longitudinal density change as “increased” or “decreased.” Density categories from all mammograms performed at a UCI Health facility on these patients were collected for this analysis, for a total of 10,342 mammograms (mean, 2.47 ± 0.82 per patient).

### 2.3. Assessment of Covariates

Each patient completed at least one electronic intake form before their screening mammography appointments, which included questions about demographics, personal and family medical history, and lifestyle factors. For patients who completed more than one electronic intake form, data for covariates were taken from the earliest intake form completed.

For race/ethnicity, participants were categorized as non-Hispanic White, non-Hispanic Asian, Hispanic, or others. Patients who indicated they were “Some other race” and those who answered “Don't know” or “Prefer not to answer” were categorized as “Other/unknown.” As few women indicated they were Black or African American, American Indian or Alaska Native, Native Hawaiian, or Other Pacific Islander ancestry, they were also categorized as “Other/unknown.”

To calculate body mass index (BMI), weight in kilograms was divided by height in meters squared. BMI was then categorized into three groups: not overweight, overweight, and obese. The body mass index categories were different for non-Hispanic Asian participants compared to the other three race/ethnicity categories based on recommendations from the World Health Organization (WHO) [27]. For the non-Asian participants, BMI categorizations <25, 25 to 30, and ≥30 were considered not overweight, overweight, and obese, respectively. For Asian participants, BMI was categorized into the three groups according to a BMI of <23, 23–27.5, and ≥27.5. Women who did not have a height or weight available from the intake form were categorized as “Missing” (*n* = 57).

Smoking status was categorized by using answers to the following questions: “Have you ever smoked regularly for 6 months or more?” and “Now, do you smoke cigarettes every day, some days, or not at all?” The categories for smoking were Never, Current, Former, and Missing. If the participant answered “No” to the first question, they were categorized as “Never.” Current smokers were considered as women who responded “Yes” to the first question and “Every day” or “Some days” to the second question. Former smokers were women who answered “Yes” to the first question and “No” to the second question. Of the women who were presented with these questions, those who answered “Don't know” to the first question or left either or both questions unanswered were considered “Missing” (*n* = 6). Questions about smoking status were added to the intake form in 2013; thus, some patients were not presented with these questions at baseline (*n* = 1030).

For the categorization of alcohol consumption (Yes/No), patients who answered “Never” to the question “How often do you have a drink containing alcohol?” were considered nondrinkers and categorized as “No.” Those who answered otherwise were considered drinkers and categorized as “Yes.” Patients who left the question blank were considered “Missing” (*n* = 22).

Patients were asked to self-report the number of days per week that they engaged in exercise considered strenuous (e.g., aerobics, aerobic dancing, jogging, tennis, swimming laps), moderate (e.g., biking outdoors, using an exercise machine like a stationary bike or treadmill, calisthenics, easy swimming, popular or folk dancing), or mild (e.g., slow dancing, bowling, golf, yoga). If they responded with an answer other than “none” for any of these types of exercises, they were asked about the usual duration in the following categories: 1 hour or more, 40–59 minutes, 20–39 minutes, or less than 20 minutes. The total weekly minutes for each of strenuous, moderate, and mild physical activity were calculated by multiplying the number of days per week times the duration (reclassified as the following: 60 minutes for “1 hour or more,” 50 minutes for “40 to 59 minutes,” 30 minutes for “20 to 39 minutes,” and 10 minutes for “less than 20”). Finally, the total weekly minutes of physical activity at all activity levels were categorized as “None” (*n* = 536), “<150 min mild, moderate, or strenuous activity per week” (*n* = 444), and “at least 150 min mild, moderate, or strenuous activity per week” (*n* = 462). If any of these questions did not have an answer, women were categorized as “Missing” (*n* = 5). Questions about exercise were added to the questionnaire in 2015; thus, a sizable number of participants were not presented with these questions at baseline (*n* = 1945).

The categorization for current hormones was based on answers to the question: “Are you currently taking any of the following hormone therapy (female hormones prescribed for women after menopause)?.” Answer choices for this were “Estrogen only pill (e.g., Premarin) Estrogen patch,” “Oral Estrogen and Progestin combination (for example, Prempro, combipatch, estrogen patch plus oral/vaginal progesterone),” “Oral Estrogen and Testosterone combination (for example, Estratest),” “Bioidentical hormones,” “Natural hormone therapy/Herbal supplements (over the counter),” “Other hormone therapy,” “Not sure what kind of hormone therapy,” and “Not currently taking any hormone therapy.” Women were categorized as “Yes” if they indicated that they were taking hormones from the answer choices. Women were classified as “No” if they did not endorse any of these answer choices or answered “Not currently taking any hormone therapy.”

### 2.4. Statistical Analysis

A univariate analysis was performed to determine whether changes in breast density were associated with breast cancer risk factors. Women with baseline mammographic densities in categories B, C, and D were included in the analyses evaluating longitudinal decreases, while women with mammographic densities in categories A, B, and C were included in the analyses evaluating longitudinal increases. Pearson's chi-squared test and ANOVA were used to detect differences in the distribution of categorical and continuous variables, respectively. Variables were selected for multivariate analysis if they were nominally significant (*p* < 0.10) in the initial univariate analyses of women who exhibited a longitudinal change in mammographic density (race/ethnicity, age at baseline, menopause age) or otherwise selected a priori based on previous literature (BMI category). Missing data were excluded from relevant statistical analyses.

Multivariate analysis, using conditional logistic regression with baseline mammographic density as strata, was performed to identify factors associated with a longitudinal increase or decrease in mammographic density. An alpha of 0.05 was used for all multivariate models. Missing data were omitted from the analyses. All data analyses were done using R, version 3.5.1 [28].

## 3. Results

### 3.1. Cohort Characteristics

Of the 3,392 women in our analytic cohort, the percent density in each category at baseline was A, 11.9%; B, 39.9%; C, 38.4%; and D, 9.8% (Table 1). The ethnic distribution of the study was 52.7% White, 23.5% Hispanic, 18.8% Asian, and 5.1% other/unknown. The average age at baseline was 61 years (range: 33 to 92), and most women reported their age of menopause was before 55 years (87.6%). Age at baseline, race/ethnicity, menarche age, parity, age at first birth, number of years since menopause, current hormone use, body mass index, smoking status, physical activity, and alcohol consumption were associated with BI-RADS density at baseline (Table 1). Overall, 7.9% of women exhibited a longitudinal decrease, 6.7% exhibited a longitudinal increase, and 85.4% did not exhibit a change in mammographic density. Stratified by race/ethnicity, we observed that a greater proportion of Asian women had dense breasts (61.6% in categories C and D) compared to White (49.4%) and Hispanic (36.7%) women (Supplementary Table 1). Asian women had the greatest proportion of “not overweight” women, while Hispanic women had the greatest proportion of obese women at baseline. In addition, Asian women had the highest proportion of nondrinkers, while White women had the highest percentage of drinkers (Supplementary Table 1).

### 3.2. Univariate Analysis of Factors Associated with Longitudinal Change in Mammographic Density

Table 2 presents the univariate analysis of women who experienced a postmenopausal increase in mammographic density. Race/ethnicity was associated with a longitudinal increase in mammographic density (*p*=0.016).  Table 3 presents the univariate analysis of women who experienced a postmenopausal decrease in mammographic density. Age at baseline and menopause age were found to be associated with a longitudinal decrease in mammographic density (age at baseline, *p*=0.003, menopause age, *p*=0.025). Race/ethnicity was nearly statistically significant (*p*=0.062); the accrual of additional patients and/or longer follow-up may clarify. The remaining variables were not statistically significantly associated with a longitudinal increase or decrease.

### 3.3. Multivariate Analysis of Factors Associated with Longitudinal Change in Mammographic Density

**Figure 2:**
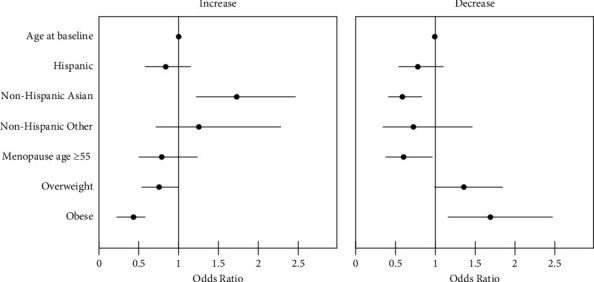
Multivariate adjusted odds ratios with 95% confidence intervals of exhibiting an increase or decrease in the BI-RADS density category.

Age at baseline, race/ethnicity, menopause age, and BMI category were included in the multivariate analyses. Asian women were more likely to exhibit a longitudinal increase in mammographic density compared to White women. On the other hand, obese women were less likely to exhibit an increase compared to normal-BMI women. Asian women were also less likely to exhibit a decrease compared to White women. Women who underwent menopause at age 55 years or later were less likely to exhibit a decrease compared to women who underwent earlier menopause. Obese women were more likely to exhibit a decrease compared to normal-BMI women (Table 4, Figure 2). The remaining variables were not statistically significant after the multivariate logistic regression.

## 4. Discussion

Consistent with previous cross-sectional studies, we observed that age, race/ethnicity, menarche age, parity, age at first birth, number of years since menopause, current hormone use, body mass index, smoking status, physical activity, and alcohol consumption were associated with BI-RADS density at baseline. However, since longitudinal change in breast density is an independent risk factor from breast density as a single measure [18, 19], we sought to obtain a better understanding of factors related to longitudinal changes in breast density. Since postmenopausal women are at higher risk for breast cancer compared to premenopausal women and would not experience fluctuations in breast density potentially associated with the menstrual cycle, our study focused on postmenopausal women.

Previous studies examining the relationship between longitudinal breast density changes and breast cancer risk have shown that breast density generally declines with age and with menopause [19, 25, 26]. Longitudinal studies that examined changes in breast density related to hormone therapy have reported increases in breast density in response to some but not all forms/doses of hormone therapy [29–32]. In addition, chemoprevention trials have shown that tamoxifen treatment is associated with decreasing breast density [33–35]. Also, surgical removal of both ovaries (bilateral oophorectomy) is associated with decreasing breast density [36, 37]. However, there have been very limited studies in which other factors were examined for potential association with longitudinal changes in breast density. One study examined longitudinal changes in breast density across the menopausal transition in 2,586 women and found that declines in breast density were greater among women with a higher premenopausal dense breast volume compared to those with a lower premenopausal dense breast volume [26]. Other breast cancer risk factors, including race, body mass index, family history, alcohol, and postmenopausal hormone therapy, had no effect on volumetric change [26]. Another study was done in a mixed group of 1,274 pre- and postmenopausal women within the multiethnic cohort. While these study participants also exhibited longitudinal decreases in mammographic density in general, women of Japanese ancestry had a 3.8% smaller decline in mammographic density for every decade of life compared to White women [19].

Our observation that Asian women were less likely to exhibit a decrease in BI-RADS density compared to White women, despite having higher BI-RADS density at baseline, was somewhat consistent with the smaller decline in mammographic density observed in Japanese women in the multiethnic cohort study. Our observation that Asian women were also more likely to exhibit a postmenopausal increase in BI-RADS density compared to White women is the first such report. Despite Asian women having a higher likelihood of having higher BI-RADS density at baseline and increasing BI-RADS density over time, Asian women have a lower risk of developing breast cancer compared to non-Hispanic White women [38–40]. This paradox may be explained in part by studies suggesting that the impact of breast density on breast cancer risk involves not only the proportion of absolute dense tissue versus nondense (fatty) tissue but also measures of absolute dense and nondense tissue [41, 42]. And although BI-RADS density scores tend to be higher in Asian women, Asian women were observed to have both smaller absolute dense areas, smaller absolute nondense areas, and lower absolute dense volume in the breast compared to Caucasian women [43, 44]. Another possible contributor to the apparent paradox of Asian women having lower breast cancer risk despite a higher likelihood of increasing in BI-RADS density is that breast cancer risk is associated with body mass index (BMI), and postmenopausal Asian women have been observed to gain less weight over time compared to women of other races [45]. Thus, the lower likelihood of Asian women experiencing a decrease in mammographic density over time is consistent with the lower extent of weight gain in Asian women and the general inverse relationship between BI-RADS density and BMI. Previous studies showed that gaining weight is indeed associated with longitudinal decreases in percent breast density due to increased amounts of nondense (fatty) breast tissue [46, 47].

In our study, we also observed that BI-RADS density in obese women was less likely to increase and more likely to decrease over time compared to women with a normal BMI. It is possible that the large amount of nondense breast tissue in obese women would make an increase in dense tissue that, if it were to occur, would be negligible in comparison and thus not increase in the BI-RADS category. We do not have an explanation for why obese women were more likely to exhibit a decrease in breast density over time. Although a decrease in breast density should be protective against breast cancer risk, a higher BMI has been consistently shown to be associated with an increased risk of postmenopausal hormone receptor-positive breast cancer, most likely due to elevated estrogen levels generated from increased adipose tissue [48, 49]. In addition, BMI has been found to be positively associated with absolute volumetric mammographic density but inversely associated with percent volumetric mammographic density [50]. Thus, the increased likelihood of decreasing BI-RADS density does not signify a decrease in absolute dense breast tissue and thus does not act as a protective factor in overweight and obese women.

Our study also suggests that women who experienced menopause at age 55 or older were less likely to show a decrease in BI-RADS density over time compared to women who underwent menopause at a younger age. A previous cross-sectional study found a positive association between age at menopause and both percent and absolute volumetric breast density [50]. To our knowledge, our findings are the first to show an association between later age at menopause and a lower likelihood of longitudinal breast density decrease. Greater age at menopause is a known risk factor for breast cancer, as is its consequence, namely, greater cumulative lifetime estrogen exposure [51, 52]. It is possible that greater cumulative lifetime estrogen exposure is reflected in a woman's breast density and perhaps exerts relative stability in breast density after menopause.

Strengths of our study include that our cohort was large and multiethnic and that we had longitudinal BI-RADS density data extracted from clinical mammographic studies. With that said, the use of BI-RADS density data assigned by human observers (clinical radiologists) may also be seen as a weakness, given that there are only four categories (as opposed to percent density, for example) and the potential for misclassification. Quantitative measurements may have been more precise, but BI-RADS density categories were readily available from the EHR and have clinical relevance, whereas quantitative breast density measurements are usually done only in the research setting. In addition, our study did not examine other measures of breast density such as absolute dense and nondense area, absolute dense and nondense volume, and percent dense volume, which are also associated with breast cancer risk [53–55]. Lastly, our study only considered factors at baseline. Longitudinal changes in BMI and other lifestyle factors may have been related to the longitudinal changes in breast density observed in this cohort.

Over a woman's lifetime, her BI-RADS breast density may change. Our data show that such changes may be influenced by race/ethnicity, BMI, and age at menopause. While we would have liked to identify modifiable factors (i.e., lifestyle factors) associated with longitudinal changes in breast density in order to guide breast cancer prevention strategies, race/ethnicity cannot be changed, and we would not want to encourage women to increase their BMI in hopes of decreasing their breast density. And while age at natural menopause cannot be modified, induced menopause as a result of surgical removal of both ovaries (bilateral oophorectomy) has been shown to be associated with decreased breast cancer risk [56–59]. However, prophylactic surgery is usually only recommended for women at the highest risk for breast cancer, for example, women with a pathogenic mutation in a breast cancer-associated gene (e.g., BRCA1). Interestingly, factors associated with a higher and lower likelihood of increasing BI-RADS breast density, namely, Asian race and obesity, respectively, are inversely associated with breast cancer risk. Meanwhile, these same factors were associated with a lower and higher likelihood of decreasing BI-RADS breast density. Our findings support the complexity of the relationships between breast density, BMI, hormone exposure, and breast cancer risk. Future studies should include more individuals, especially those from underrepresented groups, to enable stratifications by race/ethnicity, Asian ethnicities, and potentially the BMI category and other factors. Inclusion of longitudinal changes in lifestyle factors and the addition of environmental exposures may improve our understanding of why some women's breast densities increase while others' decrease and their implications on breast cancer risk and prevention.

## Figures and Tables

**Table 1:** Baseline characteristics and longitudinal BI-RADS density changes in the study cohort by baseline BI-RADS density category (*n* = 3392).

	A (*N* = 405)	B (*N* = 1352)	C (*N* = 1304)	D (*N* = 331)	Total (*N* = 3392)	*p* value
Age at baseline						<0.001
Mean (SD)	63.0 (8.8)	62.6 (8.7)	60.2 (8.6)	58.4 (8.2)	61.3 (8.8)	
Range	39–86	34–92	36–87	33–81	33–92	
Race/ethnicity						<0.001
Non-Hispanic White	213 (52.6%)	692 (51.2%)	693 (53.1%)	189 (57.1%)	1787 (52.7%)	
Hispanic	125 (30.9%)	379 (28.0%)	254 (19.5%)	38 (11.5%)	796 (23.5%)	
Non-Hispanic Asian	32 (7.9%)	213 (15.8%)	296 (22.7%)	96 (29.0%)	637 (18.8%)	
Non-Hispanic other/unknown	35 (8.6%)	68 (5.0%)	61 (4.7%)	8 (2.4%)	172 (5.1%)	
Menarche age						<0.001
<12	110 (27.6%)	254 (19.0%)	194 (15.1%)	36 (11.0%)	594 (17.7%)	
12 to 13	207 (51.5%)	675 (48.8%)	689 (50.8%)	179 (53.0%)	1750 (50.3%)	
>14	85 (21.3%)	421 (31.4%)	446 (34.7%)	104 (31.9%)	1056 (31.5%)	
Parity						<0.001
No	64 (15.8%)	202 (15.0%)	282 (21.8%)	88 (26.6%)	636 (18.8%)	
Yes	341 (84.2%)	1148 (85.0%)	1014 (78.2%)	243 (73.4%)	2746 (81.2%)	
Age at first birth						<0.001
<20	75 (22.1%)	214 (18.7%)	102 (10.1%)	23 (9.5%)	414 (15.1%)	
20 to 30	202 (59.4%)	735 (64.4%)	679 (67.0%)	125 (51.4%)	1741 (63.6%)	
>30	63 (18.5%)	193 (16.9%)	232 (22.9%)	95 (39.1%)	583 (21.3%)	
Menopause age						0.897
<55	356 (87.9%)	1179 (87.3%)	1139 (87.6%)	293 (88.8%)	2967 (87.6%)	
55 and older	49 (12.1%)	172 (12.7%)	161 (12.4%)	37 (11.2%)	419 (12.4%)	
Years since menopause						<0.001
Less than 5	54 (13.4%)	233 (17.3%)	326 (25.2%)	103 (31.5%)	716 (21.2%)	
5 or more	348 (86.6%)	1115 (82.7%)	969 (74.8%)	224 (68.5%)	2656 (78.8%)	
Current hormone use						<0.001
No	366 (90.4%)	1181 (87.4%)	1037 (79.5%)	266 (80.4%)	2850 (84.0%)	
Yes	39 (9.6%)	171 (12.6%)	267 (20.5%)	65 (19.6%)	542 (16.0%)	
BMI category (race-specific)						<0.001
Not overweight/obese	56 (14.1%)	458 (34.4%)	679 (53.0%)	260 (79.8%)	1453 (43.6%)	
Overweight	110 (27.8%)	492 (37.0%)	414 (32.3%)	52 (16.0%)	1068 (32.0%)	
Obese	230 (58.1%)	381 (28.6%)	189 (14.7%)	14 (4.3%)	814 (24.4%)	
Smoking						0.025
Never	176 (69.0%)	670 (75.1%)	764 (79.3%)	192 (78.4%)	1802 (76.5%)	
Former	63 (24.7%)	184 (20.6%)	163 (16.9%)	41 (16.7%)	451 (19.1%)	
Current	16 (6.3%)	38 (4.3%)	37 (3.8%)	12 (4.9%)	103 (4.4%)	
Physical activity						<0.001
None	78 (49.4%)	236 (41.3%)	188 (32.5%)	34 (25.2%)	536 (37.2%)	
<150 min mild, moderate, or strenuous activity per week	45 (28.5%)	170 (29.8%)	184 (31.8%)	45 (33.3%)	444 (30.8%)	
At least 150 min mild, moderate, or strenuous activity per week	35 (22.2%)	165 (28.9%)	206 (35.6%)	56 (41.5%)	462 (32.0%)	
Alcohol consumption						<0.001
Yes	166 (41.2%)	623 (46.4%)	698 (53.9%)	192 (58.0%)	1679 (49.8%)	
No	237 (58.8%)	719 (53.6%)	596 (46.1%)	139 (42.0%)	1691 (50.2%)	
Density change						<0.001
Decrease	0 (0.0%)	47 (3.5%)	143 (11.0%)	78 (23.6%)	268 (7.9%)	
Increase	68 (16.8%)	93 (6.9%)	66 (5.1%)	0 (0.0%)	227 (6.7%)	
No change	337 (83.2%)	1212 (89.6%)	1095 (84.0%)	253 (76.4%)	2897 (85.4%)	

**Table 2:** Baseline characteristics of patients who exhibited an increase or no change in the BI-RADS density category (among those with baseline BI-RADS A, B, or C).

	Increased (*N* = 227)	No change (*N* = 2644)	Total (*N* = 2871)	*p* value
Age at baseline				0.27
Mean (SD)	62.3 (9.38)	61.6 (8.69)	61.70 (8.74)	
Range	39–83	34–92	34–92	
Race/ethnicity				**0.016**
Non-Hispanic White	111 (48.9%)	1375 (52.0%)	1486 (51.8%)	
Hispanic	45 (19.8%)	674 (25.5%)	719 (25.0%)	
Non-Hispanic Asian	56 (24.7%)	454 (17.2%)	510 (17.8%)	
Non-Hispanic other/unknown	15 (6.6%)	141 (5.3%)	156 (5.4%)	
Menarche age				0.563
<12	47 (21.3%)	482 (18.4%)	529 (18.7%)	
12 to 13	105 (47.5%)	1309 (50.1%)	1414 (49.9%)	
>14	69 (31.2%)	824 (31.5%)	893 (31.5%)	
Parity				0.925
No	40 (17.7%)	473 (18.0%)	513 (17.9%)	
Yes	186 (82.3%)	2162 (82.0%)	2348 (82.1%)	
Age at first birth				0.227
<20	21 (11.3%)	345 (16.0%)	366 (15.6%)	
20 to 30	128 (68.8%)	1388 (64.4%)	1516 (64.8%)	
>30	37 (19.9%)	421 (19.5%)	458 (19.6%)	
Menopause age				0.437
<55	200 (88.9%)	2300 (87.1%)	2500 (87.2%)	
55 and older	25 (11.1%)	341 (12.9%)	366 (12.8%)	
Years since menopause				0.238
Less than 5	38 (17.0%)	533 (20.3%)	571 (20.0%)	
5 or more	186 (83.0%)	2099 (79.7%)	2285 (80.0%)	
Current hormone use				0.804
No	191 (84.1%)	2241 (84.8%)	2432 (84.7%)	
Yes	36 (15.9%)	403 (15.2%)	439 (15.3%)	
BMI category (race-specific)				0.211
Not overweight/obese	100 (44.2%)	1023 (39.4%)	1123 (39.8%)	
Overweight	76 (33.6%)	872 (33.6%)	948 (33.6%)	
Obese	50 (22.1%)	702 (27.0%)	752 (26.6%)	
Smoking				0.493
Never	115 (79.3%)	1389 (75.9%)	1504 (76.2%)	
Former	26 (17.9%)	355 (19.4%)	381 (19.3%)	
Current	4 (2.8%)	85 (4.6%)	89 (4.5%)	
Physical activity				0.244
None	44 (43.6%)	430 (38.0%)	474 (38.5%)	
<150 min mild, moderate, or strenuous activity per week	33 (32.7%)	342 (30.2%)	375 (30.4%)	
At least 150 min mild, moderate, or strenuous activity per week	24 (23.8%)	359 (31.7%)	383 (31.1%)	
Alcohol consumption				0.101
Yes	98 (43.6%)	1293 (49.3%)	1391 (48.8%)	
No	127 (56.4%)	1332 (50.7%)	1459 (51.2%)	

**Table 3:** Baseline characteristics of patients who exhibited a decrease or no change in the BI-RADS density category (among those with baseline BI-RADS B, C, or D).

	Decreased (*N* = 268)	No change (*N* = 2560)	Total (*N* = 2828)	*p* value
Age at baseline				**0.003**
Mean (SD)	59.5 (9.1)	61.2 (8.7)	61.0 (8.7)	
Range	37–89	33–92	33–92	
Race/ethnicity				0.062
Non-Hispanic White	162 (60.4%)	1327 (51.8%)	1489 (52.7%)	
Hispanic	51 (19.0%)	599 (23.4%)	650 (23.0%)	
Non-Hispanic Asian	46 (17.2%)	517 (20.2%)	563 (19.9%)	
Non-Hispanic other/unknown	9 (3.4%)	117 (4.6%)	126 (4.5%)	
Menarche age				0.278
<12	37 (14.0%)	417 (16.5%)	454 (16.2%)	
12 to 13	147 (55.5%)	1278 (50.5%)	1425 (50.9%)	
>14	81 (30.6%)	837 (33.1%)	918 (32.8%)	
Parity				0.437
No	56 (20.9%)	483 (18.9%)	539 (19.1%)	
Yes	212 (79.1%)	2068 (81.1%)	2280 (80.9%)	
Age at first birth				0.662
<20	35 (16.5%)	297 (14.4%)	332 (14.6%)	
20 to 30	130 (61.3%)	1319 (64.0%)	1449 (63.7%)	
>30	47 (22.2%)	445 (21.6%)	492 (21.6%)	
Menopause age				**0.025**
<55	246 (91.8%)	2224 (87.0%)	2470 (87.5%)	
55 and older	22 (8.2%)	332 (13.0%)	354 (12.5%)	
Years since menopause				0.844
Less than 5	61 (23.0%)	573 (22.5%)	634 (22.5%)	
5 or more	204 (77.0%)	1975 (77.5%)	2179 (77.5%)	
Current hormone use				0.483
No	219 (81.7%)	2135 (83.4%)	2354 (83.2%)	
Yes	49 (18.3%)	425 (16.6%)	474 (16.8%)	
BMI category (race-specific)				0.574
Not overweight/obese	132 (50.0%)	1173 (46.6%)	1305 (46.9%)	
Overweight	82 (31.1%)	827 (32.9%)	909 (32.7%)	
Obese	50 (18.9%)	516 (20.5%)	566 (20.4%)	
Smoking				0.234
Never	151 (77.4%)	1391 (77.1%)	1542 (77.2%)	
Former	40 (20.5%)	331 (18.4%)	371 (18.6%)	
Current	4 (2.1%)	81 (4.5%)	85 (4.3%)	
Physical activity				0.91
None	37 (34.3%)	393 (35.7%)	430 (35.6%)	
<150 min mild, moderate, or strenuous activity per week	35 (32.4%)	335 (30.5%)	370 (30.6%)	
At least 150 min mild, moderate, or strenuous activity per week	36 (33.3%)	372 (33.8%)	408 (33.8%)	
Alcohol consumption				0.538
Yes	141 (52.8%)	1292 (50.8%)	1433 (51.0%)	
No	126 (47.2%)	1250 (49.2%)	1376 (49.0%)	

**Table 4:** Multivariate adjusted odds ratios and 95% confidence intervals of exhibiting an increase or decrease in the BI-RADS density category.

	Increase		Decrease	
	OR (95% CI)	*p* value	OR (95% CI)	*p* value
Age at baseline	1.00 (0.984–1.017)	0.99	0.993 (0.977–1.009)	0.40
Hispanic	0.84 (0.576–1.225)	0.36	0.778 (0.547–1.105)	0.16
Non-Hispanic Asian	1.723 (1.212–2.449)	0.002	0.575 (0.400–0.826)	0.003
Non-Hispanic other/unknown	1.275 (0.713–2.281)	0.41	0.708 (0.346–1.450)	0.35
Menopause age 55 years and older	0.79 (0.506–1.232)	0.30	0.608 (0.377–0.979)	0.041
Overweight	0.754 (0.544–1.045)	0.09	1.352 (0.988–1.849)	0.059
Obese	0.438 (0.292–0.655)	<0.001	1.688 (1.155–2.468)	0.007

## Data Availability

Deidentified data are available on request from the corresponding authors.

## Supplementary Materials

Supplementary Materials Supplementary Table 1. Baseline characteristics of study cohort by race/ethnicity (*n* = 3392).
