# Supplementary material for: Factors Associated with Longitudinal Changes in Mammographic Density in a Multiethnic Breast Screening Cohort of Postmenopausal Women
Source: Breast J. 2023 Oct 17;2023:2794603. doi: 10.1155/2023/2794603 (PMC10597735; doi:10.1155/2023/2794603)
Supplement: Supplementary Materials — Supplementary Table 1. Baseline characteristics of study cohort by race/ethnicity (n = 3392). [file 2794603.f1.zip › Supplement.pdf]

**Supplementary Table 1. Baseline characteristics of study cohort by race/ethnicity (n=3392)**

| | Non-Hispanic White (N=1787) | Hispanic (N=796) | Non-Hispanic Asian (N=637) | Other (N=172) | Total (N=3392) | p value |
|---|---|---|---|---|---|---|
| Age at baseline | | | | | | < 0.001 |
| Mean (SD) | 62.0 (8.4) | 58.8 (8.9) | 62.5 (9.1) | 60.9 (8.6) | 61.3 (8.8) | |
| Range | 33 - 89 | 39 - 88 | 39 - 92 | 41 - 82 | 33 - 92 | |
| Menarche age | | | | | | < 0.001 |
| <12 | 319 (18.0%) | 145 (18.3%) | 77 (12.4%) | 53 (31.5%) | 594 (17.7%) | |
| 12 to 13 | 986 (55.6%) | 262 (42.3%) | 370 (46.7%) | 83 (49.4%) | 1701 (50.8%) | |
| >14 | 467 (26.4%) | 277 (35.0%) | 280 (45.2%) | 32 (19.0%) | 1056 (31.5%) | |
| Parity | | | | | | < 0.001 |
| No | 408 (22.9%) | 102 (12.8%) | 93 (14.7%) | 33 (19.2%) | 636 (18.8%) | |
| Yes | 1373 (77.1%) | 693 (87.2%) | 541 (85.3%) | 139 (80.8%) | 2746 (81.2%) | |
| Age at first birth | | | | | | < 0.001 |
| <20 | 134 (9.8%) | 230 (33.2%) | 28 (5.2%) | 22 (15.8%) | 414 (15.1%) | |
| 20 to 30 | 909 (66.5%) | 388 (56.1%) | 358 (66.3%) | 86 (61.9%) | 1741 (63.6%) | |
| >30 | 324 (23.7%) | 74 (10.7%) | 154 (28.5%) | 31 (22.3%) | 583 (21.3%) | |
| Menopause age | | | | | | < 0.001 |
| <55 | 1529 (85.8%) | 738 (92.8%) | 550 (86.5%) | 150 (87.2%) | 2967 (87.6%) | |
| 55 and older | 254 (14.2%) | 57 (7.2%) | 86 (13.5%) | 22 (12.8%) | 419 (12.4%) | |
| Years since menopause | | | | | | 0.024 |
| Less than 5 | 348 (19.5%) | 192 (24.5%) | 133 (21.0%) | 43 (25.0%) | 716 (21.2%) | |
| 5 or more | 1434 (80.5%) | 593 (75.5%) | 500 (79.0%) | 129 (75.0%) | 2656 (78.8%) | |
| Current hormone use | | | | | | < 0.001 |
| No | 1396 (78.1%) | 734 (92.2%) | 572 (89.8%) | 148 (86.0%) | 2850 (84.0%) | |
| Yes | 391 (21.9%) | 62 (7.8%) | 65 (10.2%) | 24 (14.0%) | 542 (16.0%) | |
| BMI category (race-specific) | | | | | | < 0.001 |
| Not overweight/ obese | 865 (49.0%) | 205 (26.4%) | 329 (52.4%) | 54 (32.7%) | 1453 (43.6%) | |
| Overweight | 507 (28.7%) | 286 (36.8%) | 214 (34.1%) | 61 (37.0%) | 1068 (32.0%) | |
| Obese | 393 (22.3%) | 286 (36.8%) | 85 (13.5%) | 50 (30.3%) | 814 (24.4%) | |
| Smoking | | | | | | < 0.001 |
| Never | 907 (69.4%) | 398 (79.4%) | 403 (93.9%) | 94 (78.3%) | 1802 (76.5%) | |
| Former | 334 (25.6%) | 79 (15.8%) | 18 (4.2%) | 20 (16.7%) | 451 (19.1%) | |
| Current | 65 (5.0%) | 24 (4.8%) | 8 (1.9%) | 6 (5.0%) | 103 (4.4%) | |
| Physical activity | | | | | | < 0.001 |
| None | 202 (27.9%) | 186 (54.7%) | 118 (40.8%) | 30 (33.3%) | 536 (37.2%) | |
| <150 min mild, moderate, or strenuous activity per week | 252 (34.9%) | 77 (22.6%) | 87 (30.1%) | 28 (31.1%) | 444 (30.8%) | |
| At least 150 min mild, moderate, or strenuous activity per week | 269 (37.2%) | 77 (22.6%) | 84 (29.1%) | 32 (35.6%) | 462 (32.0%) | |
| Alcohol consumption | | | | | | < 0.001 |
| Yes | 1196 (67.2%) | 241 (30.5%) | 166 (26.3%) | 76 (45.2%) | 1679 (49.8%) | |
| No | 584 (32.8%) | 549 (69.5%) | 466 (73.7%) | 92 (54.8%) | 1691 (50.2%) | |
| Baseline BI-RADS density | | | | | | < 0.001 |
| BI-RADS A | 213 (11.9%) | 125 (15.7%) | 32 (5.0%) | 35 (20.3%) | 405 (11.9%) | |
| BI-RADS B | 692 (38.7%) | 379 (47.6%) | 213 (33.4%) | 68 (39.5%) | 1352 (39.9%) | |
| BI-RADS C | 693 (38.8%) | 254 (31.9%) | 296 (46.5%) | 61 (35.5%) | 1304 (38.4%) | |
| BI-RADS D | 189 (10.6%) | 38 (4.8%) | 96 (15.1%) | 8 (4.7%) | 331 (9.8%) | |
